# Releasing Dentate Nucleus Cells from Purkinje Cell Inhibition Generates Output from the Cerebrocerebellum

**DOI:** 10.1371/journal.pone.0108774

**Published:** 2014-10-03

**Authors:** Takahiro Ishikawa, Saeka Tomatsu, Yoshiaki Tsunoda, Jongho Lee, Donna S. Hoffman, Shinji Kakei

**Affiliations:** 1 Motor Disorders Project, Tokyo Metropolitan Institute of Medical Science, Setagaya, Tokyo, Japan; 2 Frontal Lobe Function Project, Tokyo Metropolitan Institute of Medical Science, Setagaya, Tokyo, Japan; 3 Department of Neurophysiology, National Institute of Neuroscience, National Center of Neurology and Psychiatry, Kodaira, Tokyo, Japan; 4 Department of Neurobiology, University of Pittsburgh School of Medicine, Pittsburgh, Pennsylvania, United States of America; 5 Center for the Neural Basis of Cognition, University of Pittsburgh School of Medicine, Pittsburgh, Pennsylvania, United States of America; Florey Institute of Neuroscience & Mental Health, Australia

## Abstract

The cerebellum generates its vast amount of output to the cerebral cortex through the dentate nucleus (DN) that is essential for precise limb movements in primates. Nuclear cells in DN generate burst activity prior to limb movement, and inactivation of DN results in cerebellar ataxia. The question is how DN cells become active under intensive inhibitory drive from Purkinje cells (PCs). There are two excitatory inputs to DN, mossy fiber and climbing fiber collaterals, but neither of them appears to have sufficient strength for generation of burst activity in DN. Therefore, we can assume two possible mechanisms: post-inhibitory rebound excitation and disinhibition. If rebound excitation works, phasic excitation of PCs and a concomitant inhibition of DN cells should precede the excitation of DN cells. On the other hand, if disinhibition plays a primary role, phasic suppression of PCs and activation of DN cells should be observed at the same timing. To examine these two hypotheses, we compared the activity patterns of PCs in the cerebrocerebellum and DN cells during step-tracking wrist movements in three Japanese monkeys. As a result, we found that the majority of wrist-movement-related PCs were suppressed prior to movement onset and the majority of wrist-movement-related DN cells showed concurrent burst activity without prior suppression. In a minority of PCs and DN cells, movement-related increases and decreases in activity, respectively, developed later. These activity patterns suggest that the initial burst activity in DN cells is generated by reduced inhibition from PCs, i.e., by disinhibition. Our results indicate that suppression of PCs, which has been considered secondary to facilitation, plays the primary role in generating outputs from DN. Our findings provide a new perspective on the mechanisms used by PCs to influence limb motor control and on the plastic changes that underlie motor learning in the cerebrocerebellum.

## Introduction

The cerebellum generates its vast amount of output to the cerebral cortex through the dentate nucleus (DN), especially in monkeys. In fact, nuclear cells in DN generate burst activity prior to limb movement [1], [2], [3], [4], [5], [6], [7], and inactivation of DN results in cerebellar ataxia, a destruction of finely coordinated movement [8]. There are three sources of inputs to DN that may contribute to generation of the burst activity: mossy fiber (MF) collaterals, climbing fiber (CF) collaterals and Purkinje cells (PCs). MF collaterals and CF collaterals provide excitatory inputs, but neither can explain the burst activity in DN. MF collaterals are exceptionally minor in DN [9], [10], [11], [12], [13], [14], in striking contrast to the other cerebellar nuclei, i.e. the interpositus nucleus (IP) and the fastigial nucleus. Discharge of the CF (∼1 Hz) is too infrequent to explain the burst activity of DN cells. The remaining inputs from PCs are even more enigmatic because they are inhibitory and exert tonic suppression of DN cells. To explain the cause of excitation of deep cerebellar nuclear (DCN) cells in general without effective excitatory drive, there are two proposed mechanisms. First, some researchers proposed recruitment of a post-inhibitory rebound excitation [15], [16], [17], [18]. They observed a short burst of DCN cells after current-induced hyperpolarization or synchronous activation of a large number of PCs. However, there are vigorous discussions about whether the conditions required for rebound excitation are realistic in physiological conditions, especially in behaving animals [15], [16], [17], [18], [19], [20]. Second, suppression of PC activity could generate burst activity of DCN cells by disinhibition, as suggested by previous studies [13], [21], [22], [23], [24]. Indeed, Heiney et al. [25] very recently demonstrated that a transient suppression of PC activity was capable of activating DCN cells.

To address how DN cells become activated during voluntary limb movements, we compared the temporal patterns of movement-related changes in activity for PCs and DN cells recorded from the same monkeys during step-tracking movements of the wrist. If rebound excitation works, phasic excitation of PCs and a concomitant inhibition of DN cells should precede excitation of DN cells. On the other hand, if disinhibition plays a primary role, phasic suppression of PCs and activation of DN cells should be observed at the same timing. We found that a great majority of PCs showed an initial suppression of their activity prior to movement onset, while a great majority of DN cells showed an initial facilitation without a preceding suppression. In a minority of PCs and DN cells, movement-related increases and decreases in activity, respectively, developed later. Our results suggest that a decrease of inhibition from PCs, i.e., disinhibition, plays the primary role in activating DN cells. Our results further suggest that, contrary to our previous belief, suppression rather than facilitation of PCs plays the primary role in generating output from DN cells.

## Materials and Methods

### Ethics statement

All animal experimentation was conducted in accordance with the Guide for the Care and Use of Laboratory Animals (National Research Council. Washington, DC: National Academy Press, 1996) and the Guiding Principles for the Care and Use of Animals in the Field of Physiological Sciences (The Physiological Society of Japan, revised 2001). All surgical and experimental protocols were approved by the Animal Care and Use Committee of Tokyo Metropolitan Institute of Medical Science, and all efforts were made to minimize suffering.

We used three Japanese monkeys (*Macaca fuscata*, one female [monkey S] and two males [monkey M and monkey W], 6.0 kg, 8.0 kg and 7.8 kg, respectively). Animals were obtained through a government source (National BioResource Project “Japanese monkeys”). They received regular (on every weekday) veterinary checks. Each animal was housed in a cage specifically designed for macaques in an animal facility whose room temperature (18–23°C) and lighting (12-hour cycle) were controlled automatically. They were kept with other housed conspecifics and no other species. We provided animals chew toys as environmental enrichment in the cage. Animals were fed 150 g monkey biscuits once a day at 10 a.m. They also received fruit/vegetable pieces (total ∼200 g) in the afternoon. When animals were not on water control, animals had unlimited access to water through a spigot at the front of the cage. When they were on fluid control, they received water everyday regardless of performance. Body weight was measured at least once each week and the animal was taken off study if the body weight dropped below 15% of the fully hydrated weight.

**Figure 1:**
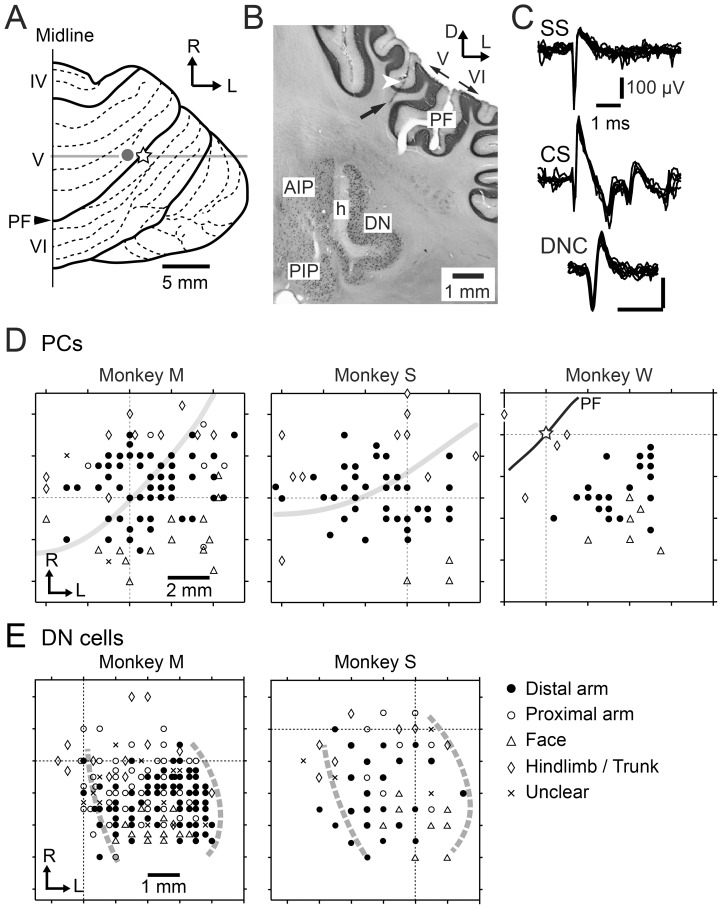
Recording sites of wrist-movement-related Purkinje cells (PCs) and dentate nucleus (DN) cells. A: Dorsal view of the right cerebellar hemisphere of monkey W. The open star indicates the center of the recording chamber. The gray dot marks the location of the electrolytic lesion indicated by the white arrowhead in B. PF: primary fissure, IV-VI: lobules IV-VI, R: rostral, L: lateral. B: Coronal section of the cerebellum of monkey W at the level of the gray line in A. The arrow indicates a recording track. The recording chamber was set at an angle to allow access to both the wrist-related cerebellar cortex and deep cerebellar nuclei (DCN). DN: dentate nucleus, AIP: anterior interpositus nucleus, PIP: posterior interpositus nucleus, h: hilum, D: dorsal, L: lateral. C: Typical examples of unit activities of simple spikes (SS, top) and complex spikes (CS, middle) for a PC and for a DN cell (DNC, bottom). D and E: Somatotopy maps of PCs for the three animals (D) and DN cells for the two animals (E). Cells with receptive fields (RFs) in distal arm (filled circles), proximal arm (open circles), face/mouth (open triangles) and hindlimb/trunk (open diamonds) are plotted. Note that all cells with RF in distal arm (filled circles) were task-related. In some cells, RF was unclear (cross marks). In D, the gray lines in the left (Monkey M) and middle (Monkey S) panels indicate locations of the PF. In the right panel (Monkey W), the open star and the PF (black line) correspond to those in A. The intersection of the two dashed lines indicates the center of the recording chamber in each animal. In E, the medial gray dashed line indicates the presumed medial edge of DN, whereas the lateral gray dashed line indicates the presumed lateral edge of DN. The medial border corresponds to the location of the axon bundle in the hilum of DN (indicated by *h* in B). The lateral border was estimated due to a lack of unit activities beyond the lines (See Materials and Methods). In both the cerebellar cortex and DN, recorded cells that had RFs in the face region were located caudal to the wrist-movement related cells, while cells that had RFs in the hindlimb/trunk were located rostrally.

A recording chamber (30 mm in diameter) was implanted in a surgical room that was specifically designed for primates using aseptic techniques and full surgical anesthesia (Ketamine, 4 mg/kg IM. and xylazine, 0.5 mg/kg IM, followed by pentobarbital sodium, initial dose  = 10 mg/kg IV, supplemented IM. as required). Animals were closely monitored prior to, during and after surgery until they could safely sit upright on their own. At the end of surgery, an analgesic was administered to the animals (Butorphanol, 0.1 mg/kg IM). The chamber was stereotaxically positioned on the hemispheric part of lobules V and VI of the cerebellum (Fig. 1A, B) ipsilateral to the trained (right) hand, based on magnetic resonance imaging (MRI). The target region was assumed to correspond to the area where arm-related PCs have been described previously [26], [27], [28], [29]. In order to record wrist-related PCs and DCN cells within a single recording chamber, we tilted the chamber laterally by 40–45 degrees from the vertical. We obtained MRI images after surgery to identify the recording area in the cerebellum. For the MRI scan, the animal was anesthetized and monitored throughout the scan and during recovery from anesthesia.

In monkey W, a small electrolytic lesion (10 µA for 10 s) was made at selected sites in the cerebellar cortex (e.g. filled gray circle in Fig. 1A and white arrow head in Fig. 1B) near the end of the recording period. Then, the monkey was deeply anesthetized with a lethal dose (75 mg/kg, IV) of pentobarbital sodium before perfusion, and perfused with physiological saline followed by 10% formalin.

### Task design of step-tracking movement of the wrist

**Figure 2:**
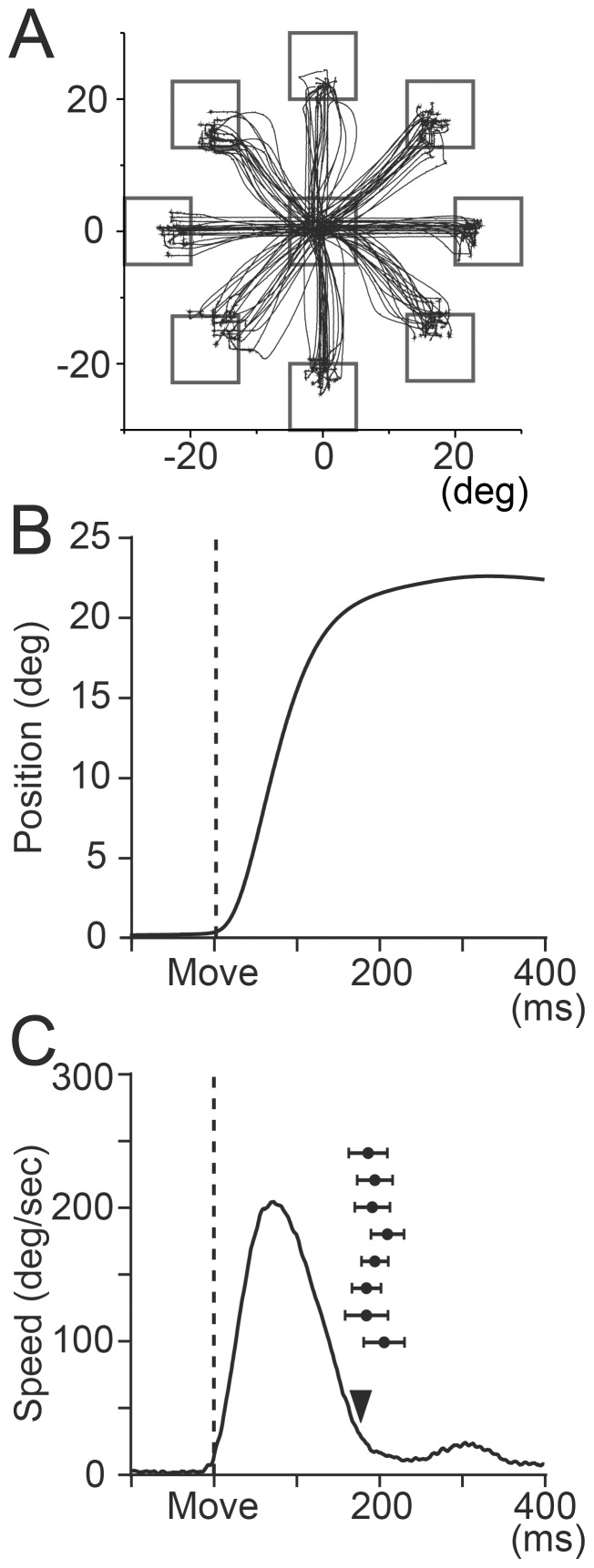
Movement kinematics of the wrist joint. A: Movement trajectories to 8 peripheral targets (10 trials for each target [square]) in the pronated forearm posture in monkey M. The target locations required 20° changes in the angle of the wrist joint. Each trace represents a single trial of movement. B: An example temporal profile of wrist angle (displacement) in a single trial. C: An example temporal profile of wrist speed in a single trial. Filled inverted triangle indicates the time of target acquisition (i.e., when the cursor moved into the target). Black circles with error bars indicate the mean ± SD of the time of target acquisition in eight movement directions (twenty trials each). Vertical dashed line labeled ‘Move’ indicates movement onset.

Details of the task were described in Kakei et al. [30]. Briefly, monkeys sat in a primate chair with their forearm supported and grasped the handle of a manipulandum. The device rotated around the two axes of wrist joint motion: flexion-extension and radial-ulnar deviation. The monkeys faced an LCD monitor and moved a small cursor that moved in proportion to the animals' wrist movements. The monkeys began the task by placing the cursor inside the central target (Fig. 2A). After a variable hold period (0.8–1.2 s), a second target (open rectangle, 8° diameter) appeared at one of eight peripheral locations evenly spaced at 45° intervals on the screen. Following a variable instruction period (1–2 s), the central target was extinguished. This served as a ‘GO’ signal that indicated to the animals to move the cursor to the peripheral target. The animals were required to complete the initial movement within 0.5 s and hold the cursor within a peripheral target for at least 0.2 s. Target locations required a 20° change in wrist angle. After 0.5 s of reaching the target, the animal obtained a drop of juice as a reward. The eight targets were presented in a randomized block design. The monkeys performed the task with the forearm in the fully pronated and/or supinated positions. Monkeys' performance was quite stable in terms of both movement kinematics (Fig. 2) and percentage of correct trials (>90%).

### Extracellular recordings and identification of cerebellar neurons

We recorded neural activity with glass-coated Elgiloy electrodes (0.8–1.8 MΩ). In order to make recordings from the deep portion of the cerebellum, we used a customized microdrive with a 40 mm range of drive (MO-95S, Narishige, Japan). We used conventional techniques to make extracellular recordings of unit activity of single cells in the hemispheric part of the cerebellar lobules V/VI and in DCN. Single unit activities were amplified (x 10,000) and band-pass filtered (150–30,000 Hz) by an amplifier (AB-611J, Nihon-Kohden, Japan), isolated with a Multi Spike Detector (Alpha Omega, Israel), and then recorded along with movement kinematics at 1 kHz for both online and offline analysis. During recordings, isolated spike waveforms of recorded cells were sampled at 20 kHz. For each cell, we recorded 5–20 trials of data for each of 8 directions in one or more forearm postures.

**Figure 3:**
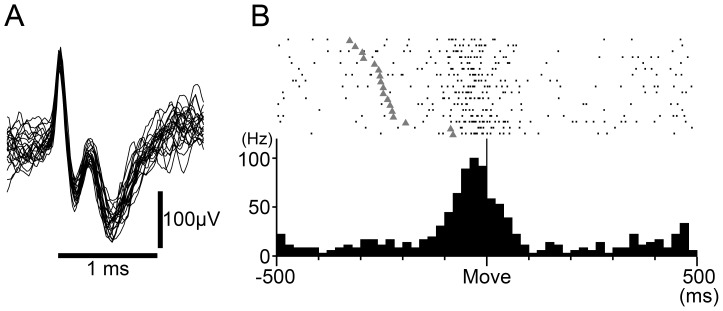
Movement-related activity of an example MF. A: A typical example of unit activity of a MF recorded at the location where task-related PCs were recorded. B: Movement-related activity of MF in A. Rasters and histogram are aligned on movement onset (Move), indicated by the solid line in the center of the histogram. Filled gray triangles in the rasters indicate the Go cue. Rasters are sorted by the timing of Go cue. Histogram bin width  = 20 ms. Similar to this example, most MFs showed a strong movement-related increase in unit activity that started before movement onset. However, onset time, duration and depth of modulation differed in each direction and among MFs.

When an electrode penetrated the tentorium cerebelli, activities of a number of putative PCs suddenly emerged. We searched for the most superficial layer of the cerebellar cortex where background noise disappeared, and considered this point as the surface of the cerebellar cortex. The depth from this point was used as a reference to identify PCs or DCN cells. PCs were identified by their location in the cerebellar cortex and the coexistence of characteristic simple spike (SS) and complex spike (CS) [27] (Fig. 1C). The occurrence of the SSs and the CSs in the same PC was identified by a silent period (>10 ms) of SSs after each CS [27], [31]. In the cerebellar cortex, activities of MFs also were recorded [32]. MF activity was identified based on their characteristic spike waveform (Fig. 3A). Because the negative after-wave represents an excitatory postsynaptic potential in granule cells (GCs) [33], it is highly likely that we recorded the MF spikes near glomeruli. To record DCN cells, we used two criteria: 1) appropriate separation from the cerebellar cortex; 2) characteristic spike waveform. After passing through the last granular layer, we advanced the electrode through the subcortical white matter for an appropriate distance (>1500 µm) before encountering cells located at edges of IP or DN (cf. Fig. 1B). The putative DCN cells were usually clustered, and they demonstrated large negative-positive spikes (e.g. Fig. 1C, DNC) with initial negativities that were usually broader than those of PCs (e.g. Fig. 1C, SS and DNC). We also required that no cells in the cluster had spike waveforms like those of PCs, MFs, or any other cell type in the cerebellar cortex. It was usually possible to distinguish between DN and IP due to the existence of the axon bundle in the hilum of DN (Fig. 1B, *h*), where we found only small positive-negative axon spikes and relatively silent background activities. In monkeys M and S, the 3-dimensional distribution of putative DN cells corresponded well with the shape of DN confirmed in monkey W by histological reconstruction. In monkey W, a small electrolytic lesion (10 µA for 10 s) was made at selected sites in the cerebellar cortex (e.g. filled gray circle in Fig. 1A and white arrow head in Fig. 1B) near the end of the recording period. The monkey was deeply anesthetized with a lethal dose (75 mg/kg, IV) of pentobarbital sodium before perfusion, and perfused with physiological saline followed by 10% formalin. After post-fixing in 30% sucrose with 10% formalin, we prepared frozen-sections of the cerebellum (50 µm thick).

### Examining receptive fields (RFs) of recorded cells

After recording unit activities, we examined the peripheral RFs of recorded cells. We used passive movements, palpation or brushing of the fingers, forearms, upper arms, shoulders, neck, chest, abdomen, back, face, and leg on both sides of the body to search for somatosensory afferent input. When a cell was activated by at least one of these stimuli, we considered the cell to have a somatosensory RF. We also searched for visual responses to directional movements of the examiner's hand in front of the animal or approach of the examiner's hand toward the animal's body. In addition, we checked whether the cells became active when the animals moved their wrist voluntarily.

### Data analysis

We analyzed the recorded data with custom-made programs on MATLAB (MathWorks, USA). To detect movement onset, we set a threshold of movement speed at 15 degrees/s for each trial. We defined the mean discharge rate before the instruction signal (−500 to 0 ms relative to the instruction signal) as the spontaneous activity level. For each recorded cell, we compared the mean discharge rate around movement onset (−200 to +300 ms relative to the onset of movement) to the spontaneous activity level using Student's *t*-test (significance set at *p*<0.01) for movements in each direction and posture. We classified cells that exhibited significant differences in discharge rate for any direction and in any posture as movement-related. The movement-related activity of those cells is the focus of this report. For each movement-related cell, we used a bootstrapping method to identify whether the cell was directionally tuned (*p*<0.05) [34].

### Calculation of onset of movement-related modulations for PCs and DN cells and display of neural activity as pseudo-colored maps

To analyze the activity of the wrist-movement-related PCs and DN cells, we processed the activity of each cell as follows. For movements in each direction and posture, we plotted a histogram of neuron activity in 20 ms bins. To display spatio-temporal patterns of activity for individual PCs and DN cells, we made pseudo-colored contour plots of the histogram. For each cell, the histograms for eight directions of movement were smoothed with a Savitzky-Golay filter (polynomial order: 3, frame size: 7), and a contour plot was generated using a standard function provided by MATLAB. In addition, to display the population activity for all movement-related PCs or DN cells in each movement direction and posture, we made pseudo-colored maps of the histograms of all PCs or DN cells according to the following procedure. First, we normalized the offset level of each histogram to zero by subtracting the average activity during the period of 160 to 220 ms before movement onset. We chose this reference time window because the earliest significant modulations of movement-related activity occurred in the next time bin (i.e., 140 to 160 ms before movement onset) for both PCs and DN cells. We smoothed the histograms with a Savitzky-Golay filter. We defined the onset of modulation as the first bin with an increase or decrease of activity of more than 10 Hz that occurred in three or more successive bins (i.e. ≥60 ms). With this method, the magnitude of the modulation changes exceeded the spontaneous firing rate by at least 2SD during the first three bins in nearly all cases (>95%). The activity in each histogram was digitized into 10 Hz-steps and mapped as a single multi-colored strip for each cell. We placed the strips for cells with initial increases on top and those for cells with initial decreases on bottom. The strips were arranged with the earliest increase at the top and the earliest decrease at the bottom. We made separate pseudo-colored maps for each monkey, movement direction and posture. Then, we converted each map into a graphical display of the numbers of cells with increases or decreases in activity during each time bin.

## Results

### Recording of PC activity

We recorded single unit activity of PCs in the lateral part of lobules V and VI of the right cerebellum (Fig. 1A, B) while monkeys used their right hand to perform wrist movements in eight different directions in two separate forearm postures: pronated and supinated [30], [35]. We found 195 PCs (85, 78, 32 in monkeys M, S, W, respectively) that displayed significant changes (*p*<0.01, Student's t-test) in SS activity during the execution period (from Go cue to movement termination) of the task. We also recorded CSs in these PCs.

Among these task-related PCs, 188 cells (80, 76, 32 in monkeys M, S, W, respectively) had somatosensory receptive fields (RFs) on the ipsilateral forearm and/or hand and fingers. These PCs, termed wrist-movement-related, are the focus of the present report. We recorded these PCs both on the surface and in the depth of the folium over a relatively wide area (6–12 mm medio-laterally, 5–6 mm rostro-caudally) (Fig. 1D). In 106/188 PCs, we collected data sets in both postures, whereas in 82/188 PCs, we collected a data set in only one posture. In total, we recorded 294 data sets from 188 PCs. The wrist-movement-related PCs demonstrated relatively high spontaneous SS activity (mean ± SD  = 46.5±16.5, 40.2±18.5, 38.0±19.5 Hz in monkey M, S, W, respectively) before the instruction signal, as reported in previous studies [26], [27], [36]. The average spontaneous SS activities showed no statistical differences among the three animals for the two postures. The remaining task-related PCs in the sample (7/195) had RFs in the upper arm, shoulder, trunk, face/mouth or leg, and were excluded from further analysis.

We also recorded a number of highly task-related MFs in the region containing wrist-movement-related PCs (Fig. 3). These MFs showed directional tuning, posture dependence and peripheral RFs that were similar to the characteristics of task-related cells in our previous recordings from M1 and the ventral premotor (PMv) area [30], [32], [35]. Therefore, it is likely that the wrist-movement-related PCs are located in the cerebrocerebellar region that receives inputs from the cortical motor areas [37], [38], [39].

### Classification of SS activity of wrist-movement-related PCs

**Figure 4:**
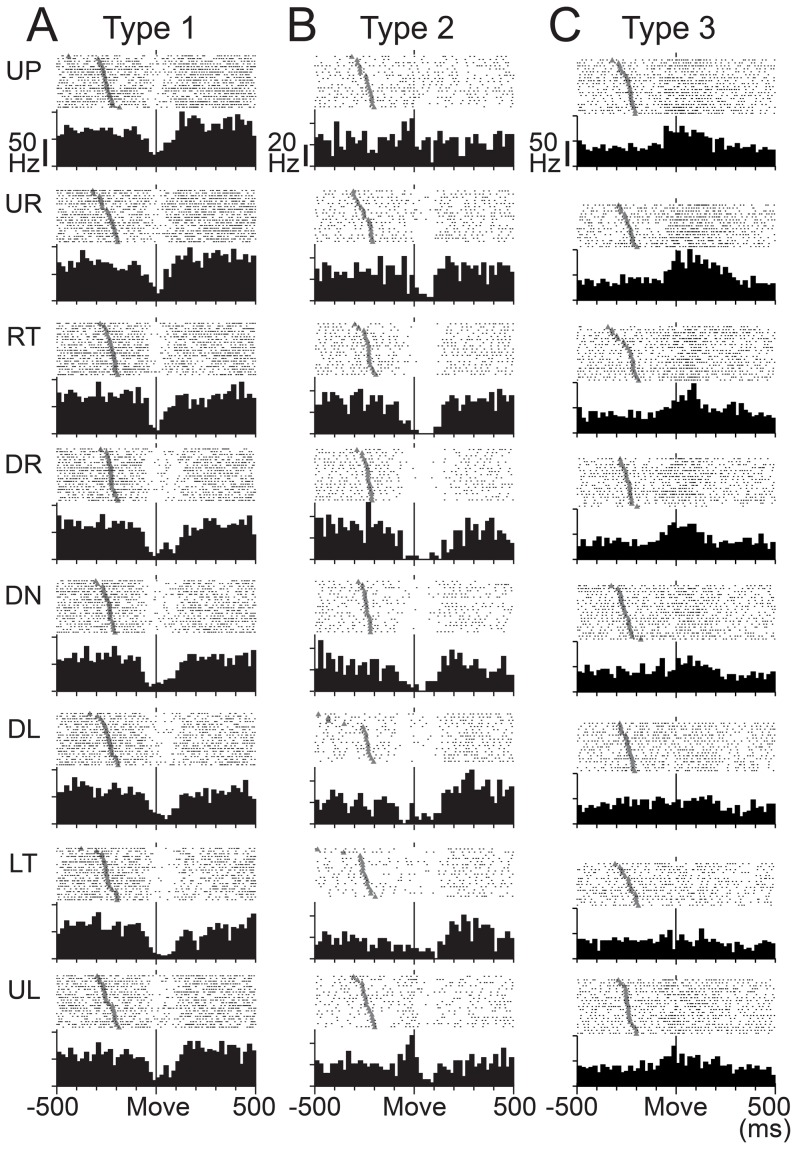
Three types of SS activity in wrist-movement-related PCs. Raster plots and histograms of SS activity for typical examples of three types of SS activity in PCs with RFs in the distal part of the arm (see Materials and Methods). SS activity recorded in the pronated posture is illustrated. PCs were classified into three types based on movement-related modulations between −200 to +200 ms relative to movement onset. All plots are aligned on movement onset (Move), indicated by the solid line in the center of each plot. In addition, all rasters are sorted by the timing of Go cue (indicated by filled gray triangles). Histogram bin width  = 20 ms. A: SS activity of type 1 PCs showed suppression before movement onset. Note that the depth and duration of suppression varied with movement direction. B: SS activity of type 2 PCs showed suppression before movement onset for some directions and facilitation for other directions. C: SS activity of type 3 PCs showed facilitation before movement onset. Note that the amplitude and duration of the facilitation varied with movement direction. Note also that the scale bars for discharge rate shown in the top row of panels differ for the three cells. UP: up, UR: upper right, RT: right, DR: down right, DN: down, DL: down left, LT: left, UL: upper left. In the pronated posture, UP, DN, LF and RT correspond to extension, flexion, radial deviation and ulnar deviation of the wrist joint, respectively.

**Figure 5:**
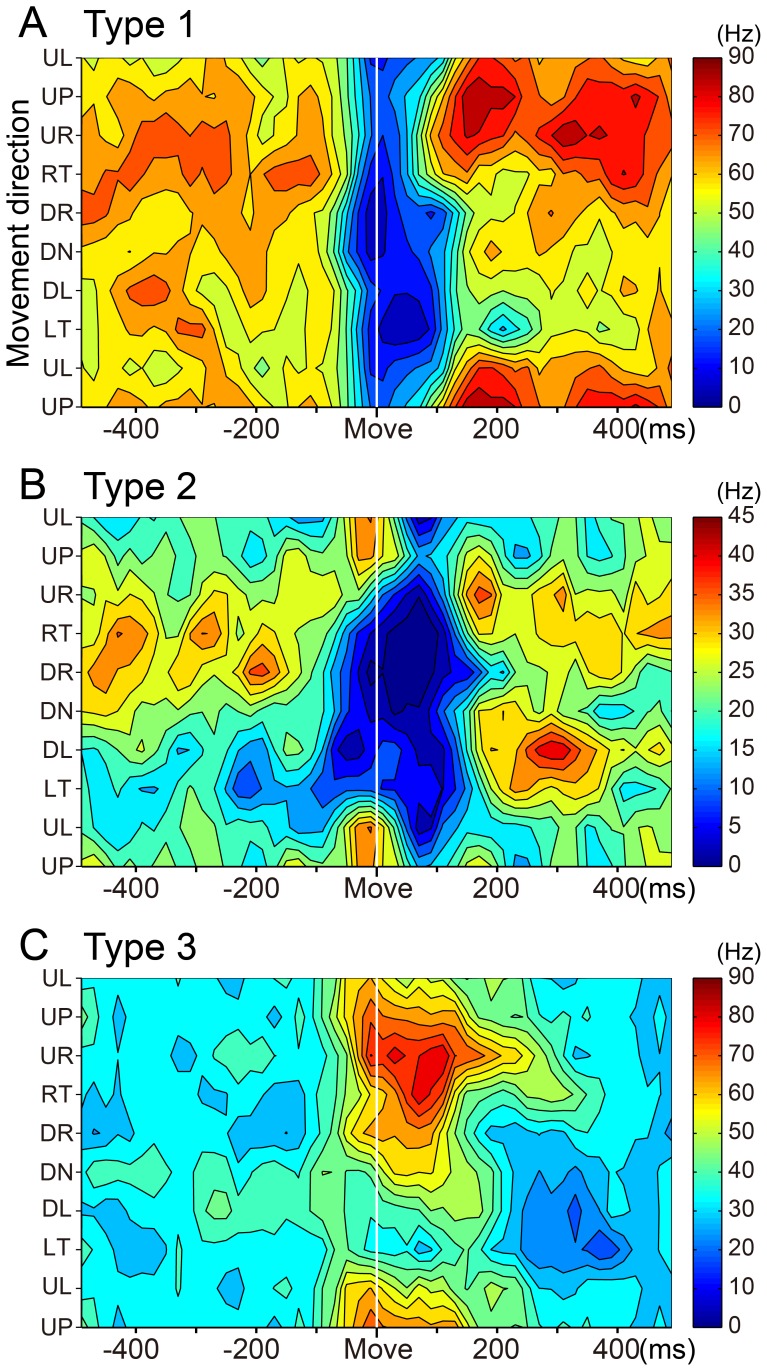
Contour plots of three types of SS activity. Spatiotemporal maps of SS activity of type 1 (A), type 2 (B) and type 3 (C) PCs in relation to 8 movement directions (the same material illustrated in Figure 4). These plots were generated with MATLAB. Color code was adjusted according to the maximum firing rate in each PC.

The wrist-movement-related PCs demonstrated a phasic modulation of their SS activity around movement onset in one or more movement directions. The high spontaneous SS activity of PCs allowed them to have bidirectional modulations, i.e., increases and/or decreases. We classified the wrist-movement-related PCs into three types based on the pre-movement modulation. Type 1 PCs showed a steep pre-movement decrease in SS activity that lasted for ∼40–200 ms in one or more directions. The representative type 1 PC (Fig. 4A) showed decreases in SS activity in all eight directions, yet the activity demonstrated directional tuning (Fig. 5A). Modulation onset and depth of suppression varied in different directions. In contrast, type 3 PCs demonstrated an increase in SS activity in one or more directions (Fig. 4C). Type 3 PCs also showed directional tuning of the onset time and amplitude of the increase (Fig. 5C). Type 2 PCs demonstrated an initial decrease in some directions and an increase in other directions (Fig. 4B and 5B). Notably, decreases were always more frequent than increases, as in this example.

**Figure 6:**
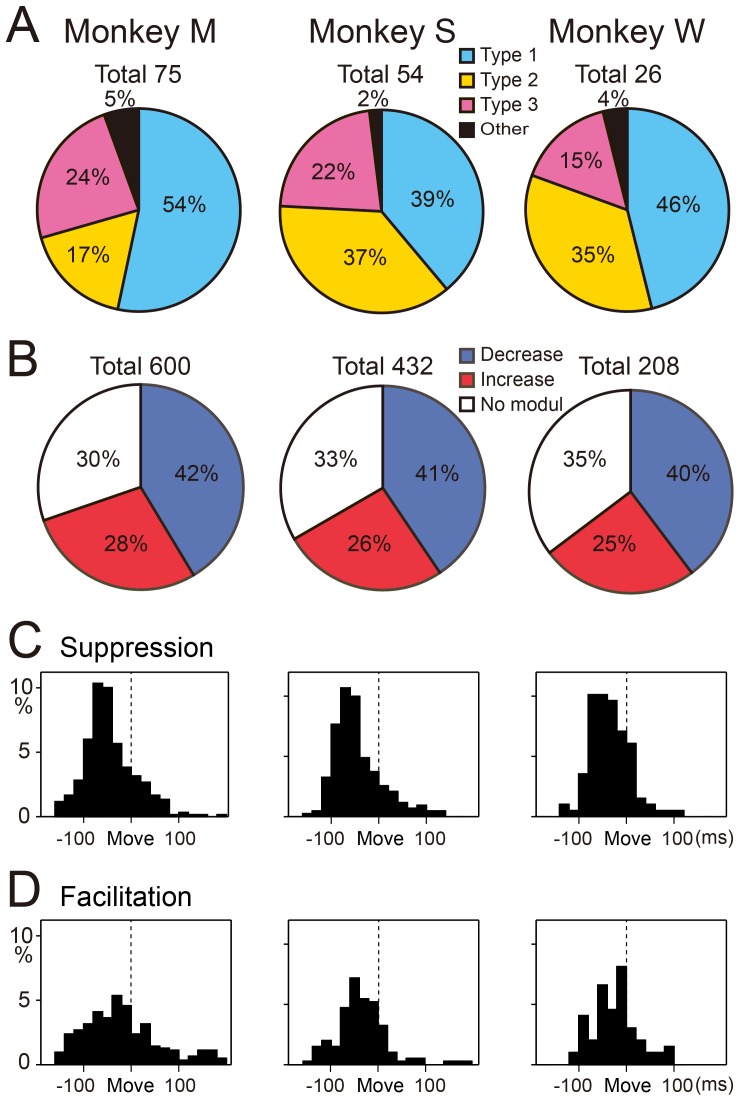
Percentages of the three types of PCs and distributions of onset latency for all suppressions and facilitations of SS activity. A: Percentages of the three types of SS activity in the pronated posture in each animal. ‘Total’ at the top of each pie chart indicates the number of cells. B: Percentages of all facilitations and suppressions among the three types of modulation in the pronated posture in each animal. ‘Total’ at the top of each pie chart indicates the number of data sets (8 directions × number of cells). C and D: Distribution of onset latencies for all initial suppressions (C) and initial facilitations (D) recorded in the pronated posture for the three animals. Mean onset latencies were significantly earlier for the suppressions (C) than for the facilitations (D) (see Table 2).

Because parallel fibers (PFs) make direct, excitatory connections with PCs, we were surprised to find that type 3 PCs (15–24%) made up the smallest proportion of our population. The type 1 PCs (39–54%) were the most common, and the type 2 PCs (17–37%) were intermediate (Fig. 6A). In the pronated posture, decreases (40–42%) in SS activity outnumbered increases (25–28%) in all three monkeys (Fig. 6B). Twenty-five PCs altered their discharge pattern between postures (from type 2 to either type 1 or 3). In general, the three types of PCs were intermingled over a wide area in the presumed cerebrocerebellum (not shown).

We examined the onset timing of the earliest movement-related modulation (i.e. facilitation or suppression) of SS activity for all PCs in each monkey, using the pronated posture (Table 1). We found that onset timing did not differ among the three types, regardless of animal (*p*>0.30, *p*>0.26, *p*>0.35 for monkeys M, S, W respectively, Mann-Whitney U test).

**Table 1:** Modulation onset of SS activity of PCs for the three animals: Earliest onset of individual PCs.

Monkey	Cell group	Mean	SD	*n*	Range
M	PC all	−97.7	41.4	71	−150 to −10
	PC inc	−101.0	51.7	24	−150 to −10
	PC dec	−95.7	33.9	47	−150 to −10
S	PC all	−89.2	31.4	53	−150 to −10
	PC inc	−88.2	30.1	22	−150 to −30
	PC dec	−90.0	33.8	31	−150 to −10
W	PC all	−74.8	30.2	25	−130 to −10
	PC inc	−78.9	24.7	9	−110 to −50
	PC dec	−72.5	33.4	16	−130 to −10

Means, SDs and Ranges are in milliseconds. PC all: all PCs. PC inc: PCs showing increased SS activity as the earliest modulation regardless of movement direction. PC dec: PCs showing decreased SS activity as the earliest modulation. *n*: numbers of PCs in individual categories. Note: sum of PC inc and PC dec equals PC all. There was no significant difference between PC inc and PC dec in all three animals (*p*>0.21, 0.80, 0.60 in monkey M, S, W, Mann-Whitney U-test).

Next, we compared the onset timing of all initial suppressions and all initial facilitations for the population of wrist-movement-related PCs in the pronated posture in the three animals (Fig. 6C, D; Table 2). The onset of suppressions occurred significantly earlier than that of facilitations for the two animals with the largest population of PCs (*p*<0.0003 and *p*<0.002 for monkeys M and S, respectively, Mann-Whitney U test) (Fig. 6C, D; Table 2). Even in the third monkey, suppressions occurred earlier than facilitations, although the difference did not reach significance due to the small number of recorded PCs (*p*<0.13, Monkey W, Mann-Whitney U test) (Table 2). We obtained comparable results for the supinated posture.

**Table 2:** Modulation onset of SS activity of PCs for the three animals: Onset of all modulations of all PCs.

Monkey	Cell group	Mean	SD	*n*	Range
M	Mod all	−32.9	67.8	560	−150 to 190
	Mod inc	−19.3	79.3	252	−150 to 190
	Mod dec	−44.0	54.3	308	−150 to 190
S	Mod all	−40.9	48.3	349	−150 to 190
	Mod inc	−35.5	39.0	137	−150 to 130
	Mod dec	−44.3	51.4	212	−150 to 190
W	Mod all	−29.9	44.8	172	−130 to 110
	Mod inc	−23.3	48.0	69	−110 to 90
	Mod dec	−34.3	42.3	103	−130 to 110

Most PCs showed modulation for two to eight movement directions. Mod all: all modulations. Mod inc: increases in modulation. Mod dec: decreases in modulation. *n*: numbers of modulations in individual categories. See also Fig. 6C and D. There was significant difference between Mod inc and Mod dec in monkey M and S (*p*<0.0003 and 0.002, respectively, Mann-Whitney U-test), but not in monkey W (*p*<0.13, Mann-Whitney U-test).

### CS activity in wrist-movement-related PCs

**Figure 7:**
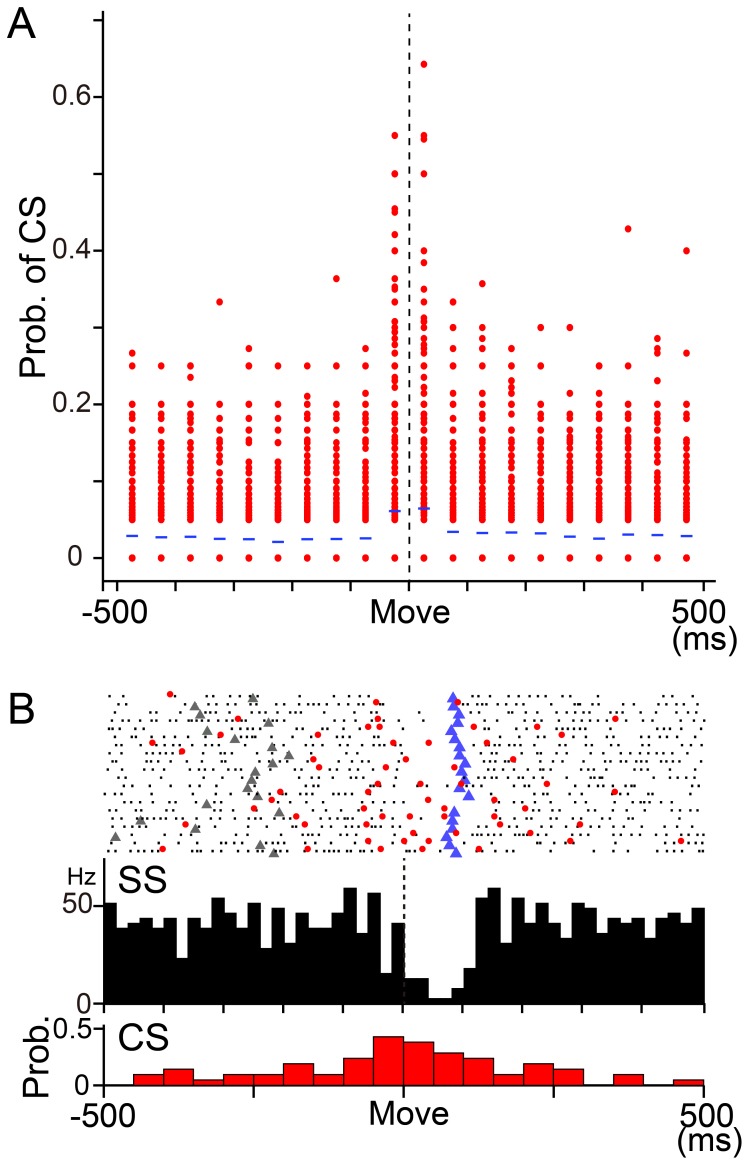
Movement-related CS activity in PCs. A: Probability of complex spike firing in 50 ms bins from −500 to 500 ms relative to movement onset in monkey M. Data from all movement directions in all PCs recorded in the pronated posture were plotted (n = 600, 8 directons ×75 PCs) in each bin. A probability of 1 means that a CS was observed in every trial in that time bin for a direction of movement in a PC. Note that the probability was 0 in more than half of PCs in each time bin, except for the bins right before and after movement onset. B: Raster plots and histograms of SS and CS simultaneously recorded in a PC. SS and CS showed a tendency of reciprocal activity around movement onset. Black dots: SS, red dots: CS, gray triangles: Go cue, blue triangles: timing of maximum velocity. Upper histogram: SS activity, 1 bin  = 20 ms. Vertical dashed line indicates movement onset. Lower histogram: CS activity, 1 bin  = 50 ms. Prob.: probability of occurrence of CS.

Mean firing rate of CSs between −500 and 500 ms relative to movement onset was about 0.6 Hz, and there was no significant difference among movement directions and postures. We calculated the probability of CS firing in 50 ms bins per trial in each movement direction in each PC by dividing the number of CSs in a bin by the number of trials (Fig. 7A). The highest probability occurred around movement onset (from −50 to 50 ms), as described in previous studies (e.g. [36]). In the example of monkey M, the probability of a CS in the time window ranged from 0 to 0.65 (mean ± SD  = 0.06±0.09). In other words, the occurrence of CSs was infrequent and inconsistent, even in the PC with the highest probability of CS. The low probability of CSs suggests that CSs did not make a reliable contribution to the activity of DN cells in our well-trained animals.

### Population activity of the wrist-movement-related SS activity in PCs

**Figure 8:**
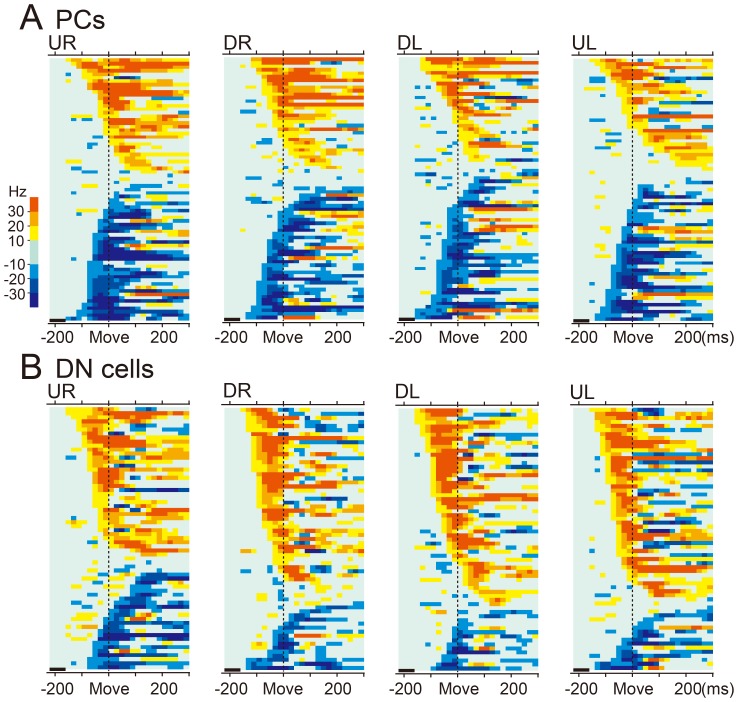
Modulation of population activity of PCs (A) and DN cells (B) in different movement directions. Pseudo-color coded summaries of modulation of SS activity of all PCs (A) and activity of all DN cells (B) for four representative targets (UR: upper right, DR: down right, DL: down left, UL: upper left) in the pronated posture in monkey M (see Materials and Methods). Each colored rectangle represents the change in SS activity or DN cell activity relative to a reference period (220 to 160 ms before movement onset, indicated by thick black bar above the abscissa of each diagram). Light/dark blue tiles represent decreases in activity, while yellow/red tiles represent increases in activity. The PCs and DN cells were arranged from top to bottom based on direction of modulation (increase or decrease) and onset time of initial modulation.

To evaluate the entire time course of wrist-movement-related output from PCs to DN cells, we analyzed the modulations of SS activity for the population of wrist-movement-related PCs in each movement direction, posture and animal (8 directions ×2 forearm postures ×3 animals  = 48 data sets). We focused on movement-related modulations during the time window from −220 to +300 relative to movement onset. Figure 8A summarizes the modulations of SS activity for 75 PCs recorded from monkey M in four representative directions (UL, UR, DL, DR) in the pronated posture. Movement-related modulations are displayed relative to the averaged activity of each PC during a reference period, 160–220 ms before movement onset (Fig. 8A, *thick black bars above abscissa*). Light/dark blue tiles represent decreases in SS activity, while yellow/red tiles represent increases in SS activity, respectively.

**Figure 9:**
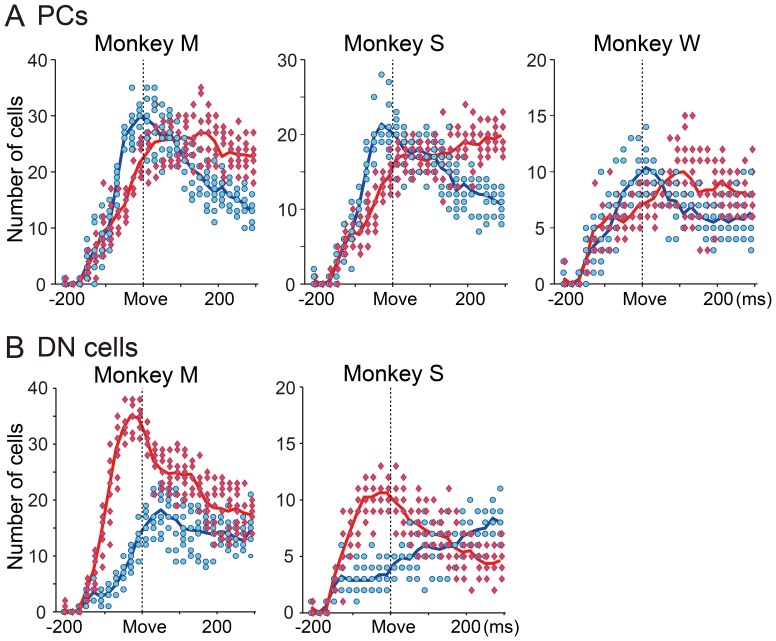
Temporal patterns of recruitment of all PCs and DN cells with facilitation or suppression of activity for eight different directions. A: PCs. B: DN cells. In each time bin (20 ms), blue dots represent the number of PCs or DN cells with decreased activity, and red diamonds represent the number of PCs or DN cells with increased activity for eight different directions of movement in the pronated posture in each animal. Blue and red lines indicate mean numbers of blue dots and red diamonds in individual bins, respectively.

In general, PCs with increases in SS activity and those with decreases began to show significant changes in their firing rate about 160 ms before movement onset (Fig. 9A, blue and red lines). However, shortly thereafter (about 140 ms before movement onset), the graphs show a striking divergence between the numbers of PCs with decreases and those with increases. Before movement onset (from −100 to 0 ms), PCs with decreases in SS activity outnumbered those with increases for all four directions (Fig. 8A, *decreases*: *increases*, UL 28:19; UR 29:22; DL 34:22; DR 31:27). Indeed, the majority of datasets showed a dominance of PCs with decreases in SS activity in this early time period, regardless of animal (Fig. 9A) (20/24 datasets: 7/8 directions in monkey M, 7/8 in monkey S, 6/8 in money W). In contrast, 140–240 ms after movement onset, PCs with increases in SS activity outnumbered those with decreases for all four directions (Fig. 8A, *decreases: increases*, UL 20:29; UR 18:33; DL 17:23; DR 20:27) and for all animals (Fig. 9A). Overall, the PCs with movement-related decreases in SS activity dominated before and for a short period after movement onset, whereas the PCs with increases in SS activity began to dominate ∼100 ms after movement onset (Fig. 9A). We obtained comparable results for the supinated posture. Considering the brief velocity pulse and short movement duration of the wrist movements (Fig. 2B and C), we suggest that PC activity before and just after movement onset is associated with execution of the movement, whereas later activity is associated with stopping the movement in the target and maintaining the wrist position at the end of movement.

The difference in the temporal patterns of suppression and facilitation of PCs suggests that the outputs of the PCs to individual DN cells may not cancel each other, even if they converged onto a common DN cell. Because PCs exert a strong inhibition on DN cells, the initial dominance of PCs that show decreased activity may provide excitatory drive to their target DN cells before and just after movement onset. The subsequent dominance of PCs with increased activity may modulate or suppress the preceding activation of DN cells.

### Wrist-movement-related activity of DN cells

**Figure 10:**
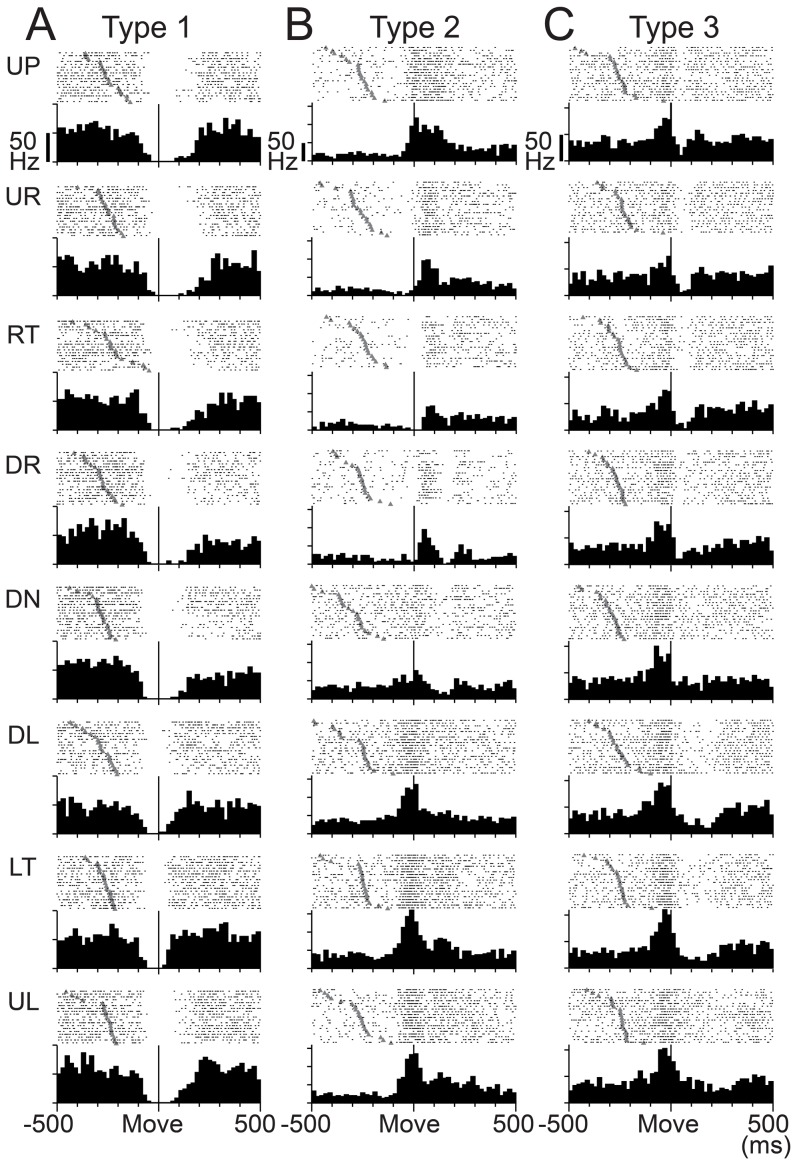
Three types of activity in wrist-movement-related DN cells. Raster plots and histograms of typical examples of three types of activity in DN cells with RFs in the distal part of the arm. Neuron activity recorded in the pronated posture is illustrated. DN cells were classified into three types based on movement-related modulations between −200 to +200 ms relative to movement onset. This figure uses the same conventions as Figure 4. A: Activity of type 1 DN cells showed suppression before movement onset. B: Activity of type 2 DN cells showed suppression before movement onset for some directions and facilitation for other directions. C: Activity of type 3 DN cells showed facilitation before movement onset. Note that the scale bars for discharge rate shown in the top row of panels differ for the three cells.

In order to examine the proposed mechanisms for cerebellar outputs, we recorded the activity of DCN cells from monkeys M and S during the step-tracking task. We found 192 DCN cells (142 in monkey M, 50 in monkey S) that showed significant task-related modulations. We estimated that these DCN cells were recorded mostly from DN. This estimate is based on their location lateral to the axon bundle in the hilum of DN (Fig. 1B, *h*), along with the chamber maps of the recorded DCN cells (Fig. 1E) and the MRI images for both monkeys (see Materials and Methods). We first excluded fifty-nine DN cells that had RFs in the upper arm, shoulder, face, or leg or were unresponsive to somatosensory stimuli. The remaining 133 task-related DN cells responded strongly to both passive and self-initiated movement of the wrist. Eighty-four of these 133 DN cells showed significant movement-related activity starting before movement onset. Below, we focus on the activity of these 84 “wrist-movement-related” DN cells (65 in monkey M and 19 in monkey S). These wrist-movement-related DN cells demonstrated spontaneous discharges (mean ± SD  = 32.4±12.1 Hz, range 8–61 Hz, n = 84), as seen in the examples in Figure 10. The largest modulation of their activity around movement onset (−200 to +200 ms) was comparable with previous studies in DN and IP (mean ± SD  = 66.1±25.9 Hz, range 14–147 Hz) [1], [2], [3], [4], [5], [6], [7].

### Classification of activity of wrist-movement-related DN cells

**Figure 11:**
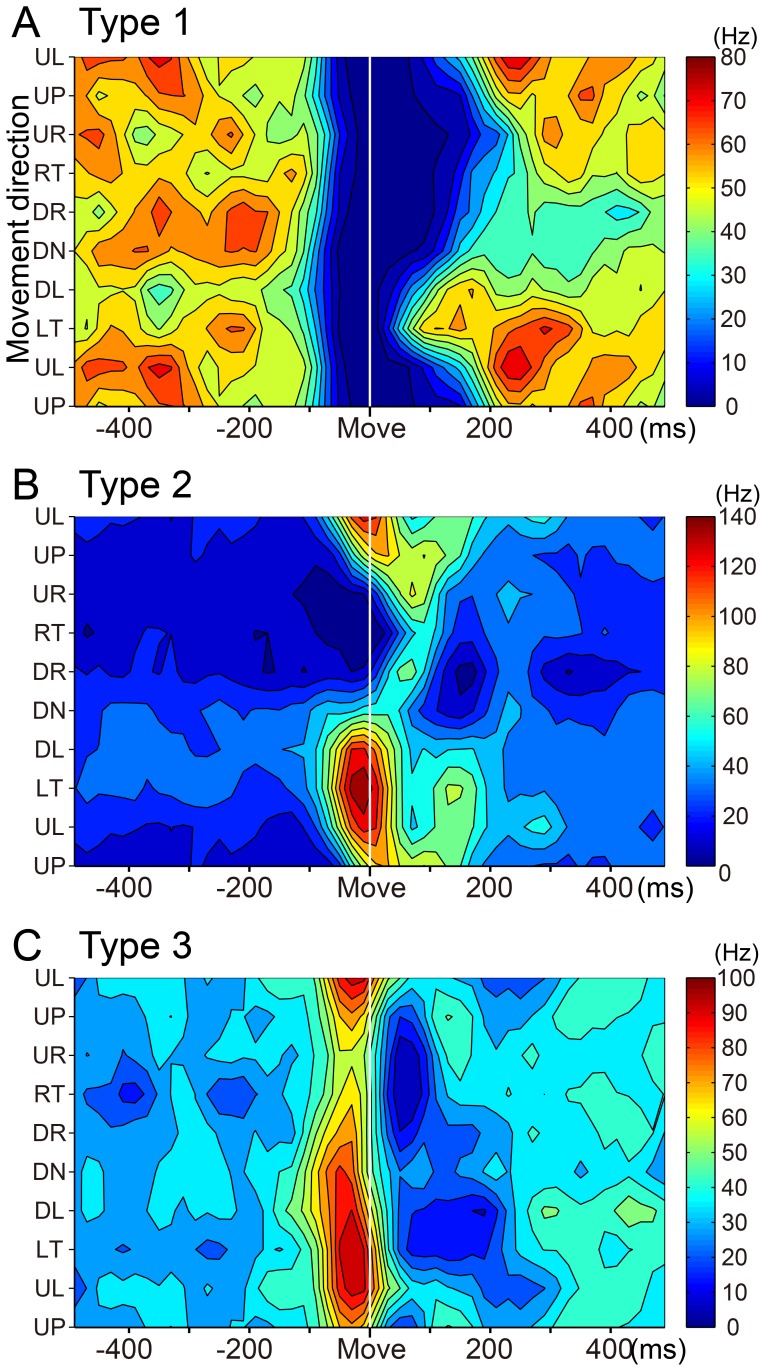
Contour plots of three types of DN cell activity. Spatiotemporal maps of neuron activity of type 1 (A), type 2 (B) and type 3 (C) DN cells in relation to 8 movement directions for the same cells illustrated in Figure 10. These plots were generated with MATLAB. Color code was adjusted according to the maximum firing rate in each DN cell.

We classified the wrist-movement-related DN cells into three types using the same categorization as for the SS activity of PCs. Type 1 DN cells showed a steep decrease in activity that lasted for ∼60–200 ms in one or more directions (Fig. 10A). In contrast, type 3 DN cells demonstrated a phasic increase in activity in one or more directions (Fig. 10C). The type 1 and 3 DN cells demonstrated directional tuning of onset time and modulation amplitude (Fig. 11), as we observed in PCs (Fig. 5). Type 2 DN cells demonstrated an increase in some directions and a decrease in other directions (Fig. 11B). Notably, increases were always more frequent than decreases, as in this example. Significantly, most phasic increases in activity of DN cells occurred without evidence of a prior suppression, even on a single-trial basis (Fig. 10B, C). In addition, most DN cells with a movement-related pause of activity did not show a subsequent rebound excitation (Fig. 10A).

**Figure 12:**
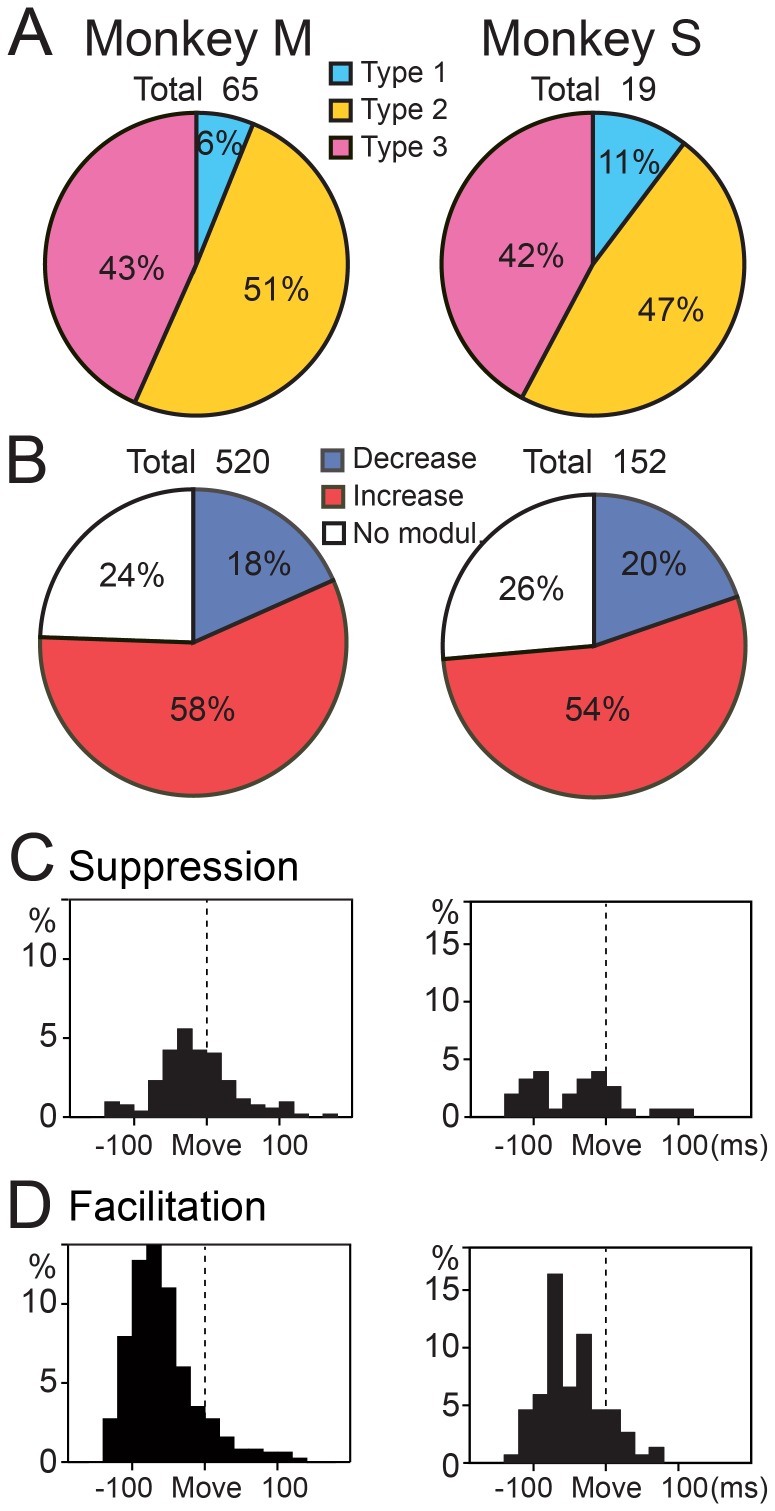
Percentages of the three types of DN cells and distributions of onset latency for all suppressions and facilitations of DN cell activity. A: Percentages of the three types of DN cell activity in the pronated posture in monkeys M and S. ‘Total’ at the top of each pie chart indicates the number of cells. B: Percentages of all facilitations and suppressions among the three types of modulation in the pronated posture in each animal. ‘Total’ at the top of each pie chart indicates the number of data sets (8 directions × number of cells). C and D: Distribution of onset latencies for all suppressions (C) and facilitations (D) recorded in the pronated posture for the two animals. Mean onset latencies were significantly earlier for the facilitations (D) than for the suppressions (C) (see Table 4).

We observed a striking difference in the proportions of the 3 neuron-types for the DN cells (Fig. 12A), compared with the PCs (Fig. 6A). The type 2 DN cells (51, 47%) and type 3 DN cells (43, 42%) were quite common, whereas the type 1 DN cells were infrequently observed (6, 11%) in both monkeys (Fig. 12A). Overall, initial increases (58, 54%) outnumbered initial decreases (18, 20%) in the pronated posture for both monkeys (Fig. 12B). In general, the three types of DN cells were intermingled over a wide area (not shown). We analyzed onset timing of the earliest modulations and all initial suppressions and initial facilitations in the two animals, as summarized in Fig. 12C, D and Tables 3 and 4. In striking contrast to the corresponding results for PCs (Fig. 6C, D), we found that the facilitations occurred earlier than the suppressions in DN cells (*p*<5.34×10^−20^ and *p*<0.09 for monkeys M and S, respectively, Mann-Whitney U test, Fig. 12C, D). We obtained comparable results for the supinated posture. The finding that facilitations are more numerous and show earlier recruitment than suppressions in DN cells strongly suggests that the primary and major wrist-movement-related output from DN is facilitation.

**Table 3:** Modulation onset of activity of DN cells for the two animals: Earliest onset of individual DN cells.

Monkey	Cell group	Mean	SD	*n*	Range
M	DNC all	−93.1	29.4	65	−150 to −10
	DNC inc	−95.9	26.6	51	−150 to −30
	DNC dec	−82.9	37.3	14	−130 to −10
S	DNC all	−92.1	38.2	19	−150 to −10
	DNC inc	−85.0	37.6	16	−150 to −10
	DNC dec	−130	—	3	−130

The same conventions as in Table 1. DNC all: all DN cells. DNC inc: DN cells showing increased activity as the earliest modulation regardless of movement direction. DNC dec: DN cells showing decreased activity as the earliest modulation. There was significant difference between DNC inc and DNC dec in monkey S (*p*<0.002, Mann-Whitney U-test), but not in monkey M (*p*<0.27, Mann-Whitney U-test).

**Table 4:** Modulation onset of activity of DN cells for the two animals: Onset of all modulations of all DN cells.

Monkey	Cell group	Mean	SD	*n*	Range
M	Mod all	−44.7	53.9	484	−150 to 190
	Mod inc	−58.5	47.6	335	−150 to 190
	Mod dec	−13.6	54.6	149	−130 to 130
S	Mod all	−56.4	35.9	128	−150 to 110
	Mod inc	−62.4	35.9	90	−150 to 50
	Mod dec	−42.1	35.6	38	−130 to 110

Most DN cells showed modulation for two to eight movement directions. Mod all: all modulations. Mod inc: increases in modulation. Mod dec: decreases in modulation. See also Fig. 12C and D. Mod inc occurred earlier than Mod dec in monkey M and S (*p*<5.3×10^−20^ and 0.09, respectively, Mann-Whitney U-test).

### Population activity of the wrist-movement-related DN cells

To assess the time course of modulation of activity in the wrist-movement related DN cells, we performed the same analysis as that used for the SS activity of PCs (Fig. 8B). Using the time window of −220 to +300 ms relative to movement onset, we evaluated the modulations of activity for the population of wrist-movement-related DN cells separately for each movement direction, posture and animal (8 directions ×2 forearm postures ×2 animals  = 32 data sets). Figure 8B summarizes the modulations of activity for 65 DN cells recorded in monkey M in four representative directions (UL, UR, DL, DR) in the pronated posture. The graphs in Fig. 9B show the numbers of DN cells with decreases in activity (blue lines)(Fig. 8B, light/dark blue tiles) and the numbers of DN cells with increases in activity (red lines)(Fig. 8B, yellow/red tiles) separately in each 20 ms time bin. In general, DN cells with increases in activity and those with decreases in activity began to show significant changes in firing rate about 160 ms before movement onset. Significantly, DN cells with increases in activity strongly outnumbered those with decreases in activity throughout the entire movement-related period (Fig. 9B). The dominance of DN cells with increases in activity was greatest before and just after movement onset, and decreased ∼100 ms after movement onset (Fig. 9B).

### Relative timing of modulation of the wrist-movement-related PCs and DN cells

We compared the *earliest* modulation for individual PCs and DN cells in monkeys M and S (Tables 1 and 3). We found that the timing of the earliest change in activity for PCs and DN cells did *not* differ significantly in either monkey (*p*>0.85 in monkey M and *p*>0.67 in monkey S, Mann-Whitney U test). We also specifically compared the onset times for the earliest suppressions of PCs and the earliest facilitations of DN cells. The time of onset for the earliest suppressions of PCs and the earliest facilitations of DN cells did not differ significantly in either monkey (*p*>0.43 in monkey M, *p*>0.56 in monkey S, Mann-Whitney U test) (Tables 1 and 3). We obtained comparable results for the supinated posture. Thach [36] also demonstrated that there was no significant difference between onset of PCs and DN cells during arm movements.

### Correspondence between the cerebellar cortex and DN

The PCs and DN cells reported in this study demonstrated significant wrist-movement-related activity and clear somatosensory RFs in the ipsilateral forearm and/or hand and fingers. The PCs were distributed as a large cluster over lobules V and VI (Fig. 1D). The DN cells also were distributed as a large cluster (Fig. 1E). We found PCs and DN cells with RFs in the leg rostral to wrist-movement-related cells and those with RFs in the orofacial region caudal to wrist-movement-related cells (Fig. 1D, E). Thus, both PCs and DN cells displayed a rostro-caudal arrangement of leg, forelimb and orofacial regions, as described in physiological studies [40], [41] and an anatomical study [39]. Furthermore, task-related MFs recorded adjacent to the PCs showed pre-movement modulations of activity that were similar to those of task-related cells in M1 and PMv [30], [32], [35]. The pre-movement modulations of MFs support our view that we recorded PCs mainly from the cerebrocerebellum rather than from the spinocerebellum, where PCs project to IP. Overall, we found a strong functional and morphologic correspondence between the regions in the cerebellar cortex and DN where we recorded wrist-movement-related cell activities.

## Discussion

How do DN cells become active under strong inhibition from PCs? To the best of our knowledge, the present study is the first to address this question by comparing the patterns of activity of PCs and DN cells in awake animals performing limb movements. We found that the majority of PCs in the cerebrocerebellum were suppressed prior to the onset of rapid wrist movements (Figs. 4A and 6A). At the same time the majority of DN cells were activated without prior suppression (Figs. 10C and 12A). We showed further that the activity of PCs with movement-related suppressions developed earlier than the activity of PCs with movement-related facilitations (Figs. 8A and 9A). Notably, we observed the opposite temporal pattern for the movement-related DN cells, namely, movement-related facilitations became predominant prior to movement-related suppressions (Fig. 8B and 9B). Taken together, our observations suggest that the movement-related activation of DN cells occurs when they are released from tonic inhibition by PCs, i.e. disinhibition. Below we discuss the implications of these results.

### Selection of DN

DN is not the only cerebellar nucleus that makes a critical contribution to limb motor control. IP is also important for limb movement [6]. Nevertheless, our selection of DN rather than IP was very critical for this study. IP receives strong MF collateral inputs from various sources such as lateral reticular nucleus [14], external cuneate nucleus [42] or spinal cord [11]. In contrast, DN has long been recognized to have exceptionally weak MF collateral inputs [9], [10], [11], [12], [13], [14], [43]. For instance, less than 10% of MFs from PN send sparse collaterals to DN [13]. Overall, the MF collateral input cannot be the primary determinant of activities of DN cells. Therefore, we focused on the relationship between DN and the corresponding part of the cerebellar cortex.

### Comparing activity of PCs and DN cells during step-tracking movements of the wrist

In a prior study, inactivation of DN made step-tracking arm movements ataxic [8]. Therefore, DN, more generally the cerebrocerebellum, is essential for coordination of the fast and precise movements employed in this study [30], [35]. Furthermore, we designed the experiment optimally to compare activities of PCs and DN cells in the same monkey. First, we trained the animals to perform a wide range of wrist movements with stereotyped movement kinematics (Fig. 2), so that we could analyze reproducible neuron activities (e.g., Figs. 4 and 10). Second, we adjusted the location and angle of the recording chamber (see Materials and Methods) to maximize the chance of recording from PCs and DN cells related to movements of the distal forelimb (see Figs. 4 and 8 in Lu et al. [39]). Indeed, in a recent study [32], we reported that a great majority of MFs recorded in the same region of the cerebellar cortex in the same animals (Monkey M and S) showed delay period and/or pre-movement period activities (Fig. 3) that are comparable to those observed in M1 or PMv neurons [30], [35]. This result strongly suggests that we recorded PCs in the region of the cerebrocerebellum that is connected to M1 or PMv [38], [39]. Overall, the present study is suitable for making precise comparisons of the temporal patterns of movement-related activities in PCs and DN cells.

### Activation of DN cells by disinhibition

Previous authors reported that DN cells become strongly active prior to the onset of limb movements in monkeys [1], [2], [3], [4], [5], [6], [7], as confirmed in this study (Fig. 10). Three mechanisms have been proposed for the excitation of DN cells: 1) collateral inputs from MFs; 2) rebound excitation of DN cells; 3) disinhibition of DN cells. We examined the potential contributions of these mechanisms to DN cell activity by comparing the temporal patterns of movement-related modulations for PCs and DN cells in monkeys performing step-tracking wrist movements.

If activation of DN cells arises mainly from collaterals of the ponto-cerebellar projection, then bursts of activity in DN cells should precede modulation of PCs, as was observed during visually guided saccades in the fastigial oculomotor region and the oculomotor vermis [44]. However, we did not find a significant difference between the earliest modulation of PCs and the earliest modulation of DN cells, as Thach [36] reported previously. In addition, previous morphologic data suggest that the contribution of MF collaterals may be small within DN [13], [45], [46]. These studies found that DN cells receive a relatively weak projection from a minority of the ponto-cerebellar MFs. Moreover, terminations of the ponto-cerebellar collaterals were located on the distal ends of dendrites of DN cells, which would minimize their synaptic efficacy [47]. Indeed Holdefer et al. (2005) [48] clearly demonstrated that excitatory inputs from MF collaterals were undetectable in DN cells without blocking the strong inhibitory PC input. Therefore, it is unlikely that MF collaterals play a primary role in generating the pre-movement burst of DN cells. Nevertheless, our data may suggest a contribution of collateral input to the pre-movement burst in DN cells. We observed a small dominance of decreases over increases in PCs (Figs. 8A and 9A) and a more marked dominance of increases over decreases in DN cells (Figs. 8B and 9B). This difference is consistent with an additional excitatory input to DN cells that counteracts inhibition by PCs. Therefore, MF collaterals may play an assistive role in activating DN cells, as was demonstrated for IP cells *in vivo* [49].

Second, activation of DN cells may be triggered by a post-inhibitory rebound excitation after a phasic increase of suppression from PCs, as shown *in vitro* [15], [16], [17] and *in vivo* [16], [18], [20] experiments with artificial stimuli. However, in the present study performed in behaving monkeys without artificial stimuli, we found that a great majority of PCs exhibited strong suppression of activity before movement onset (Figs. 4A, B and 8A), at the same time that a minority of PCs showed facilitation of activity (Figs. 4C and 8A). Most DN cells showed activation without evidence of prior suppression (Figs. 8B, 10B, C). In addition, most DN cells with a movement-related pause of activity did not show a subsequent rebound excitation (Figs. 8B and 10A). A few DN cells showed a rebound-like activity, but this occurred only *after* movement onset (Fig. 8B). Thus, in behaving monkeys, prior suppression is not required to generate a burst activity in DN cells, and even a complete halt of discharge is not enough to trigger the rebound mechanism of DN cells (Fig. 10A). In agreement with our observations, Alvina et al. [19] demonstrated that rebound firing of DCN cells is not frequently observed even *in vitro*. We note also that rebound firing has been demonstrated in IP, but not in DN where we recorded neurons. Considering the differences in input-output organization of these two nuclei, it is possible that DCN cells in DN and IP differ in their response to inputs from the cerebellar cortex. Overall, we conclude that rebound excitation did not make a major contribution to the generation of the movement-related burst activity of DN cells in our experimental conditions and recording location.

We also evaluated the possibility that synchronized CSs may initiate rebound excitation of DN cells [16], [20] through synchronous activation of a population of PCs in our preparation. The firing probability of CSs showed a slight increase in two 50 ms time bins before and after movement onset (Fig. 7A), as was described previously (e.g. [36]). However, a peak firing probability above the chance level (i.e. *p* = 0.5) was extremely rare in either bin, regardless of PC and movement direction. The average firing probability even in these two time windows was only 0.06 and 0.07. Overall, the level of CS activity during our task was far below the synchrony achieved by direct or indirect stimulation of the inferior olive.

Recently, Person and Raman [50], [51] demonstrated the possibility of another type of synchronous PC activity to activate DCN cells. This mechanism does not require a preceding increase in activity of individual PCs. Rather, it involves synchronous activity of a number of PCs that project to an individual DN cell. We cannot exclude that this mechanism may contribute to activation of DN cells in awake, behaving animals. However, a great majority of the task-related PCs demonstrated a strong decrease in activity before movement onset (Figs. 4A and 9A), which would minimize the efficacy of synchronous PCs. Overall, synchronous activity of PCs may contribute at best to a fraction of the pre-movement activation of DN cells.

Third, activation of DN cells may be triggered by disinhibition [13], [21], [25]. Both PCs and DN cells are known to have unusually high spontaneous activities [27]. Indeed, we observed high baseline activity in the PCs (∼40 Hz) and DN cells (∼30 Hz) recorded in this study. This high activity in PCs would exert a strong tonic suppression of the high intrinsic activity of DN cells. If this is the case, then suppression of PC activity prior to movement onset, as observed in the present and many previous studies [26], [29], [31], [36], [52], would disinhibit the target DN cells and release their intrinsic activity. Indeed, DCN cells can be reliably activated by a brief suppression of their inhibitory inputs [53]. Furthermore, transient suppression of PC activity induced prompt activation of DN cells followed by a movement [25]. In this study, a great majority of DN cells generated movement-related bursts of activity without a preceding suppression that is required for rebound excitation. Taken together, our observations suggest that disinhibition plays a primary role in activating DN cells. On the other hand, it should be emphasized that disinhibition and rebound excitation are not mutually exclusive. That is, a ‘*reduction of inhibition*’ triggers excitation of DN cells in either case. As suggested by Bengtsson et al. [20], some ionic mechanisms and conductance machinery underlying rebound excitation could assist in speeding up the response of DCN cells to the fast reduction in SS activity of PCs that we observed. The two mechanisms may be distinguishable by careful examination of the temporal features of modulation of PCs and DN cells. However, disinhibition might activate DN cells with only a synaptic delay (∼1 ms), which would be undetectable in our experimental setup. On the other hand, a rebound response requires a strong inhibition of longer duration (>10 ms, e.g. Hoebeek et al. [16]) before activation of DN cells. Future experiments comparing activities of PCs and DN cells with identified connectivity will be needed to confirm our conclusion that DN cells are most likely activated by disinhibition.

Why has suppression of SS activity attracted relatively little attention so far? For a long time, it was assumed that the *primary* modulation of SS activity was an increase. Previous studies reported increases of SS activity in behaving monkeys for arm movements [26], [36], [54], [55], [56] and eye movements [44], [57], [58]. Nevertheless, all of these studies also noted a considerable or even comparable number of PCs with decreases in SS activity. Harvey et al. [56] and Espinoza and Smith [55] reported that 40% and 44% of the arm-movement-related PCs showed decreases in SS activity, respectively. Similarly, Coltz et al. [54] reported that 41% of PCs showed a decrease in SS activity for increases in arm velocity. In the present study, about 55% of all initial modulations of wrist-movement-related SS activity were suppressions (Fig. 6B) in all three monkeys. Overall, for studies that analyzed arm movements, the emphasis on increases in SS activity may have resulted largely from a historical focus on excitation, rather than on a difference in observations between prior experiments and ours.

The early, strong suppression of SS activity observed in this study is likely to be mediated by molecular layer interneurons (INs), especially basket cells [59]. It is commonly thought that monosynaptic activation of PCs should precede disynaptic inhibition of PCs by a few milliseconds, but our results indicate that the disynaptic pathway may be recruited faster than expected. We suggest that multiple mechanisms may shorten the delay in the disynaptic pathway, as reviewed by Jörntell et al. [59]. First, there is evidence that EPSPs from PFs are large and have a fast rise time in INs, which facilitates a very rapid spike initiation in INs [60], [61], [62]. Second, axon terminals of INs wrap around somata and initial segments of PCs [63] and provide exceptionally effective inhibition of PCs [64], [65]. Taken together, these features suggest that the disynaptic inhibitory pathway is optimally designed to provide fast, strong and precisely timed inhibition of PCs and result in burst activity in DCN cells via disinhibition [25]. Indeed, Ebner and his colleagues clearly demonstrated that stimulation of a PF beam induced disynaptic inhibition of PCs at a *lower* threshold than was needed for direct excitation of PCs (Fig. 7 in Gao et al. [66]). The lower threshold of PC inhibition is consistent with our suggestion that the inhibitory pathway is recruited earlier in behaving monkeys.

### Asymmetric processing through parallel pathways in the cerebellar cortex

**Figure 13:**
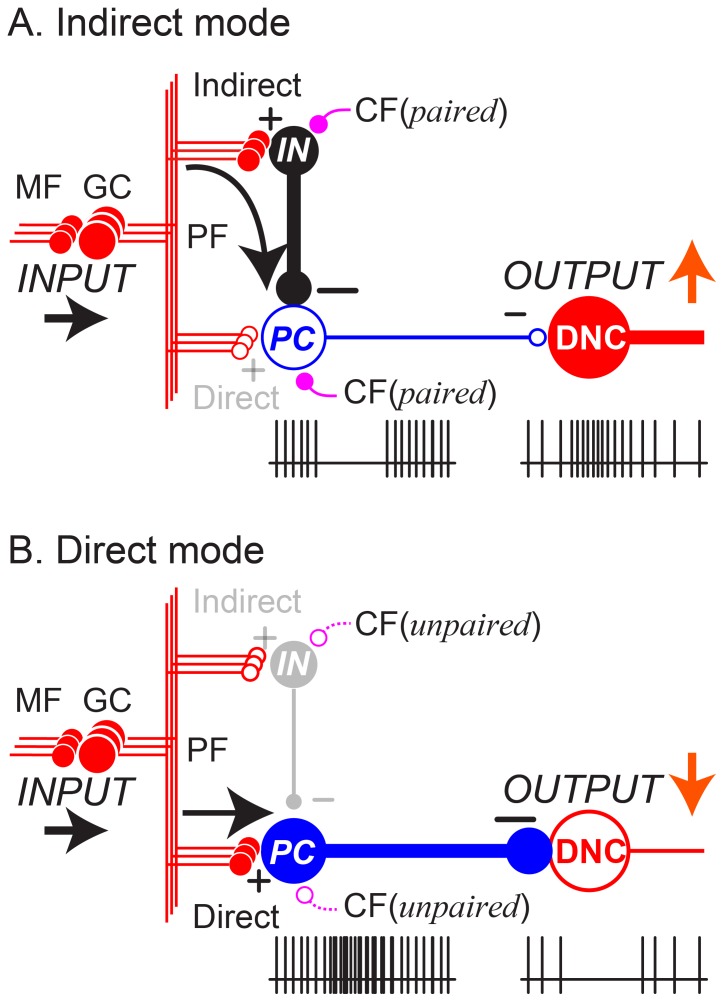
Parallel pathways in the cerebellar cortex determine two modes of DN cell (DNC) output. A. Indirect mode. B. Direct mode. This is a summary diagram of the functional organization of the cerebellum supported by this study. In the cerebellar cortex, mossy fiber (MF) inputs (INPUT) are relayed by granule cells (GCs) and are processed differently through two parallel pathways, an indirect pathway (Indirect) and a direct pathway (Direct). In the indirect pathway, parallel fiber (PF) inputs activate interneurons (INs) that suppress PCs. Because PC activity provides a tonic suppression of DNCs, the suppression of PC activity facilitates DNCs through disinhibition (A, OUTPUT↑). In the direct pathway, PF inputs excite PCs directly. Because PCs are inhibitory, their activation suppresses the DNCs (B, OUTPUT↓). The balance between the two pathways determines the final output patterns of individual PCs. In this way, inhibitory PCs are able to exert bidirectional effects on DNCs and enable a variety of cerebellar output patterns. CF: climbing fiber. Paired: CF activity paired with PF inputs. Unpaired: CF activity unpaired with PF inputs. Pluses (+) represent excitatory synapses, and minuses (−) represent inhibitory synapses. Note that, in this diagram the PC and the IN share inputs from the same PFs. This is meant to show the basic connectivity of the cerebellar cortex in the simplest form. In reality, a PC and an associated IN may or may not have common PF inputs.

MF inputs to the cerebellar cortex are relayed by GCs and then processed in two *parallel pathways* to PCs (Fig. 13) [67]. The *direct pathway* activates PCs directly, while the *indirect pathway* uses inhibitory INs to suppress PCs. Dean et al. [67] proposed that the parallel pathways work in a cooperative way to suppress or facilitate activity of PCs in the cerebellar cortex. Based on our observations, we extend their idea to explain the role of the parallel pathways for generation of cerebellar output from DN. The parallel pathways provide two modes for transforming MF inputs to DCN outputs though PCs (Fig. 13). Because PC outputs are inhibitory, excitation of PCs through the direct pathway suppresses their target DN cells, and suppression of PCs through the indirect pathway facilitates their target DN cells. We found that movement-related suppressions of SS activity dominated before movement onset and movement-related excitations dominated after movement onset in our population of PCs (Figs. 8A and 9A). We propose that the indirect pathway plays the leading role in initiating cerebellar outputs for limb movements.

In this study, we focused on modulation of SS activity of PCs. Nevertheless, we need to discuss CF inputs and relevant synaptic plasticity to understand how the two modes of SS modulation are acquired (Fig. 13). It is generally accepted that CF inputs have reciprocal effects on PF inputs to INs and PCs [61], [68], [69], [70], [71]. Stimulation of a PF input *paired* with CF activity induces LTP of PF-IN synapses and LTD of PF-PC synapses. Therefore, it is likely that decreases in SS activity are developed by repetitive *paired* activation of PF and CF inputs. In contrast, stimulation of a PF input *unpaired* with CF activity induces LTD of PF-IN synapses and LTP of PF-PC synapses. Therefore, it is likely that increases in SS activity are developed by repetitive *unpaired* activation of PF and CF inputs. For this reciprocity in development of plasticity to occur, PCs showing decreases in SS activity and PCs showing increases in SS activity concurrently should be innervated by distinct CFs with a low or even negative correlation.

Several prior studies have provided strong support for the reciprocity between CS activity and SS activity of a PC. For instance, CS activity and SS activity in individual PCs showed reciprocal activities during ocular following [72], smooth pursuit [73] and limb movements [31], [36], [74] (see also Fig. 7B). Furthermore, during saccadic adaptation, adaptive changes of CS activity and SS activity demonstrated a negative correlation [75].

### Consideration of the operating principle of the cerebrocerebellum

The cerebellum has long been regarded as “neural machinery” designed to process input information in some unique and essential manner [76]. In this regard, it is most important to determine the primary mode of operation of the inhibitory PC in the cerebrocerebellum. The PC determines how movement-related information is represented, processed and output from DCN, thereby defining the functional organization of the cerebrocerebellum. To our knowledge, previous studies gave greater attention to facilitation of the PC as the *primary* modulation and understated suppression of the PC, despite repeated observations of suppression of SS activity in voluntary arm movements [26], [29], [31], [36], [52]. The bias toward facilitation of SS activity is understandable because electrical PF stimulation induced an initial excitation and later inhibition of PCs in anesthetized animals [77]. In the present study, we have clearly demonstrated that the primary modulation of the PC in the cerebrocerebellum is suppression of SS activity for execution of wrist movements in behaving monkeys. As a result, we propose that output from the DN is primarily triggered by disinhibition. Our results provide a new perspective on the mechanisms used by PCs to influence limb motor control and on the plastic changes that underlie motor learning in the cerebellum.
